# On the genetic and environmental sources of social and political participation in adolescence and early adulthood

**DOI:** 10.1371/journal.pone.0202518

**Published:** 2018-08-24

**Authors:** Anna E. Kornadt, Anke Hufer, Christian Kandler, Rainer Riemann

**Affiliations:** 1 Department of Psychology, Bielefeld University, Bielefeld, Germany; 2 Department of Psychology, MSB Medical School Berlin, Berlin, Germany; Boston University, UNITED STATES

## Abstract

Political participation (POP), social participation (SOP), and political interest (PI) are important indicators of social status and social inequality. Previous studies on related trait differences yielded genetic and environmental contributions. However, focusing on adult samples, classical twin designs, and convenience samples often restricts parameter estimation and generalizability, and limits the understanding of age differences. We investigated sources of variance in POP, SOP, and PI in late adolescence and early adulthood with an extended twin family design (ETFD). We analyzed data from over 2,000 representative German twin families. Individual environments not shared by family members reflected the major source of variance for all variables, but genetic influences were also pronounced. Genetic effects were mostly higher for young adults, whereas effects of twins’ shared environment were significant in adolescence. Our study deepens the understanding of the interplay between genetic and environmental factors in shaping differences in young persons’ integration in society.

## Introduction

Individuals’ participation in the political decision process (political participation, POP) and participating in societal institutions like volunteer organizations (social participation, SOP) are indispensable features and pillar stones of modern democratic societies [1]. By voting for their preferred party or candidate or by participating in rallies and protests, people attempt to influence the direction of policies in their countries or municipalities [2]. More individually, POP for example fosters perceptions of political efficacy [3, 4]. Furthermore, the integration into social groups increases social networks and support. SOP is also instrumental in gaining opportunities, skills, and knowledge for further civic engagement, and promotes a sense of integration in the community [5–8]. So even though POP and SOP focus on somewhat different behaviors and outcomes, they both indicate whether people are included, can partake and participate in or profit from social and political processes. Therefore, it is highly relevant to understand the underlying factors of individual differences in POP and SOP, as well as political interest (PI), which represents the intrinsic motivation and a prerequisite to POP [2, 9, 10].

Since people differ strongly in interests and engagement in politics or social groups, a large body of research has tried to identify what sources determine individual differences in SOP, POP, and PI. Environmental factors such as parental and peer socialization or socioeconomic resources have been considered, since parents and their children show pronounced similarities in those characteristics [11, 12]. This seems like a strong argument for the existence of variation in parental socialization of SOP, POP, and PI. However, focusing exclusively on environmental explanations of individual differences does not take into account that children are not only subjected to their parents as role models and the environments provided by the parents, but also inherit their parents’ genes. Thus, individual differences between families and similarity between parents and children within families might be due to both socialization and (or) genetic transmission. Moreover, genetic and environmental influences are not independent, but show patterns of correlation and interaction: For example, the parents’ genetic makeup is related to the environments they provide for their offspring (passive gene-environment correlation, e.g. [13]). Genetic and environmental explanations of individual differences can thus be seen not as mutually exclusive but rather intertwined and integrative [11, 14–17]. Considering the role of genes and their interplay with the environment is necessary to fully understand the etiology of individual differences in POP, SOP, and PI. In support of this claim, previous studies that investigated genetic and environmental variance components have found evidence for both influences (for overviews, see e.g., [18, 19]).

Heritability estimates (i.e., the degree to which individual differences are due to genetic variance) for POP typically range between .30 and .60 [14, 16, 17, 20] in adult samples. Regarding the environmental contribution, environmental factors not shared by family members acting to decrease the similarity of family members (i.e., non-shared environmental effects) were strongest, whereas shared environmental effects (i.e., factors making people from the same family more similar) are considerably smaller or even negligible. Similar results were found for individual differences in acts of civic engagement, volunteering, charitable giving, and social activities [15, 21]. However, we are not aware of behavior genetic studies that have looked at the genetic and environmental contributions to the variance in SOP operationalized as integration in a variety of social groups. This concept of SOP taps into a special form of social participation aimed at social integration per se and not necessarily at civic duty or social responsibility compared to previous studies interested in civic engagement and volunteerism in a narrower sense [15, 21]. Individual differences in PI also seem to be primarily due to genetic differences and variation in individual environmental experiences, with only small effects of the shared environment [14, 22], leading Klemmensen et al. [9] to the conclusion that PI might be a dispositional trait and part of a person’s political personality.

Since gene expression and societal circumstances of an individual’s SOP and POP develop and change over the life span, considering age differences is crucial when investigating genetic and social transmission within families [23]. This is especially true for adolescence (roughly the age from about 12 to 18 years [24]) and young adulthood (roughly the age from 18 years until the late 20ies [25]), since they can be considered as *“developmental periods of substantial flux in gene expression and emerging environmental opportunities”* ([26], p. 424). During adolescence, children most commonly live together with their parents and siblings, and therefore, are more likely directly influenced by environments shared by family members, such as the living environment, parental expectations and norms, or the household’s socioeconomic status. Thus, individual differences in adolescent political activities and interests might be more strongly influenced by parents and siblings. During emerging adulthood, children have more freedom in their life conduct, and thus shared environmental influences on individual differences may decrease [19]. This may be accompanied by an increased expression of young adults’ individuality including their genetic predispositions, and thus empirically, an increase in population estimates of genetic variance can be observed, since young adults are attracted to specific environments that match their heritable traits (active gene-environment correlation; [13]). Indeed, the meta-analysis by Bergen et al. [26] shows that for most behavioral phenotypes, genetic variance increases from adolescence to young adulthood. These age differences might be especially relevant for our variables of interest, since adolescence and young adulthood are critical periods in the formation of political and civic behavior [27]. Furthermore, emerging adulthood also marks a period when people come of age and thus have the legal opportunities to participate in more and different political and social activities (for social activities such as sports participation, see also [28]).

Previous behavior genetic studies on traits related to SOP, POP, and PI that take age or developmental differences into account indeed found that genetic and environmental contributions to variance depend on participants’ age: Eaves and colleagues [29] investigated a large sample of twins aged 9 to 75 years and showed that the shared environment contributed to a large degree to the variance in conservatism under the age of 20, whereas genetic effects were most important in adulthood. Hatemi and colleagues [23] reported that whereas genetic influences on individual differences in political attitudes (liberalism-conservatism) were negligible in childhood (around age 10), and environmental effects (shared and non-shared) most pronounced in adolescence (around age 17), genetic effects became substantial starting in young adulthood (around age 21). Investigating social attitudes (religiousness and conservatism) in an adoption study of adolescents aged 12 to 15 years, Abrahamson and colleagues [30] also found strong influences of the shared environment and fewer genetic influences during this age period. Besides these findings concerned with more attitudinal aspects of political and social traits, age group comparisons for SOP, POP, and PI are absent.

The goal of our study was to use a sophisticated behavior genetic design and a large sample to illuminate the genetic and environmental sources of individual differences in SOP, POP, and PI in two cohorts of individuals in critical developmental periods and compare the resulting estimates. Previous genetically informative studies on SOP, POP, and PI have mostly relied on classical twin designs (CTD). Including only the twins in the analyses means that there is not enough information in the data to estimate genetic and environmental transmission between generations, specific age-dependent environmental sources of siblings’ similarity, and gene-environment interplay. Including siblings and parents of the twins allows for the estimation of these effects and thus helps us to better understand refined genetic and environmental sources and their interplay [31]. Our study thus applies this method to study the sources of individual differences in SOP, POP, and PI. It allows us to disentangle genetic from environmental effects due to direct parental transmission, different aspects of siblings’ environments, and environments specific for different family members, as well as effects due to passive gene-environment correlation.

Drawing on previous, established literature, we assume strong genetic contributions to variance in POP and PI. Furthermore, we include a measure of SOP for which behavior genetic results have to our knowledge not been reported. The inclusion of these three distinct but related variables broadens the focus of previous research towards a more comprehensive picture on political and social integration and participation. This is especially relevant, since we can base our analyses on a large population-based sample from Germany representing the whole spectrum of socioeconomic status in the country and considerably less biased in terms of over-representing highly educated, high-income families [32]. In addition, we were also interested in age-group differences in genetic and environmental influences and hypothesize that genetic variance will be larger for young adults compared to adolescents, indicating a more important role of active gene-environment correlation in adulthood.

## Materials and methods

### Sample

We used data from the TwinLife study, a study of twins and their families in Germany. TwinLife is designed to understand the development of social inequalities over the life course, and captures a variety of constructs related to life chances in childhood, adolescence and early adulthood. It combines a prospective multi-cohort cross-sequential design with an extended twin family design (see analyses section), and the sample is based on a national probability sample. Since there is no central twin register in Germany, same-sex twin pairs from four birth cohorts (5-, 11-, 17-, and 23-year old at the first measurement point) were identified via local registry offices. The sophisticated sampling and recruitment procedure is described in detail in [33]. The goal was to obtain a large sample from each birth cohort within a broad range of the population, and about equal proportions of MZ and DZ twins. Thus, a two-step national probability-based sampling procedure was implemented. First, a sample of communities within Germany was drawn to generate the addresses. Then, for each of the interesting cohorts, individuals with the same sex born at the same day and living at the same address were identified (a slightly different procedure was used for the oldest cohort, due to the low probability that they still live at the same household, for details, see [33]). From these samples of addresses, subsamples for each cohort were drawn and the twins and their families were contacted by a social survey research institute. If they agreed to participate, they were visited in their homes by a trained interviewer, who distributed the research instruments.

Due to the sampling method and design, the TwinLife sample is representative of German families that have multiple children, for example with respect to region of residence and the size of the communities where the household is located. The sample is also representative in terms of German citizenship status on the household level, highest educational and occupational status of parents in the household and monthly net equivalent household income in euros, and therefore covers the whole range of socioeconomic structure in Germany [33] Further details regarding the TwinLife rationale, the study design, recruitment strategies, data collection, and zygosity determination can be found in [32, 33]. The TwinLife data set is open source and can be obtained for research projects (http://dx.doi.org/10.4232/1.12665, see also http://www.twin-life.de/en). The TwinLife study was approved by the Ethics Committee of the German Association of Psychology.

For the current analyses, we included cross-sectional data from the oldest two cohorts of twins, born 1990–1993 (Cohort 23, C23) and 1997/1998 (Cohort 17, C17) respectively, and if available, their parents and siblings. This selection was made since detailed data on SOP and POP was only available for participants aged 13 and older (Since PI was assessed already from age 10, the sample includes some younger siblings for this variable). Our sample consisted of *N* = 2042 full twin pairs, this sample size provides sufficient power for our intended analyses [34]. We also included biological mothers and fathers, as well as one full sibling into our design if available, thus the maximum of included participants per family was five. A detailed sample description of the subsample is presented in Table 1.

**Table 1:** Sample description.

	*N*			Age	
	Total	% female	Complete pairs	*M* (*SD*)	Range
MZ twins					
C17	996	56.2	498	17.01 (0.36)	16–18
C23	1048	59.4	524	23.06 (0.83)	21–25
DZ twins					
C17	1122	58.3	561	17.01 (0.32)	16–18
C23	918	56.6	459	23.02 (0.85)	21–25
Siblings					
C17	494	46.4	-	18.43 (4.77)	12–33
C23	453	51.2	-	24.60 (5.09)	12–40
Mothers					
C17	1035	100	-	47.70 (4.55)	34–63
C23	955	100	-	52.58 (4.61)	41–69
Fathers					
C17	914	0	-	50.53 (5.03)	36–74
C23	822	0	-	55.24 (5.27)	42–79

Note. MZ = monozygotic; DZ = dizygotic; C17 = younger cohort; C23 = older cohort.

### Measures

#### Political participation

POP was assessed with three items that capture different activities indicative of political participation: *“Which of the following activities did you take part in within the last 12 months*?*”* 1) *Taken part in a political meeting/a discussion event/a demonstration*; 2) *Taken part in an online-petition/a signature collection*; 3) *Boycotted a company or products for political or ethical reasons or on environmental grounds*, *e*.*g*. *did not buy products or avoided them* (cf. [20]). All items were answered with yes or no and combined into an additive index, with higher values indicating higher POP [15]. Cronbach’s alpha across all family members was .584, which is consistent with results for similar scales in the previous literature [11] and acceptable for such a brief scale summing rather heterogeneous behaviors subsumed under the multifaceted construct of POP [1].

#### Political interest

PI was assessed with one item *“Generally speaking*, *how interested are you in politics”*. Participants rated this question on a four-point scale (1 –not interested at all, 2 –not strongly interested, 3 –strongly interested, 4—extremely interested).

#### Social participation

To measure participants SOP, we assessed the amount of activity in different groups or clubs; items were adapted from the German Youth Survey [35]. *“In the following you see a number of groups one can be active in*. *Please indicate to what extent you are active in these respective groups*. 1) *Sports association or club;* 2) *Choir/music or theatre group or similar;* 3) *Church or religious group;* 4) *Trade union/professional association/student council;* 5) *Voluntary fire and rescue services/technical relief association THW/DLRG German Lifesaving Association etc*.; 6) *Local history association/Citizens association/Shooting club ("Schuetzenverein");* 7) *Other associations/federation”*. One item concerning membership in a political organization was not included in the analyses, in order not to confound measures of SOP and POP. For each group, participants had to indicate how often they were active by answering on a four-point scale with 1 –every week, 2 –every month, 3 –less than once a month, 4 –never. Answers were combined to a composite score, with lower values representing more frequent activities.

### Extended Twin Family Model analyses

**Fig 1:**
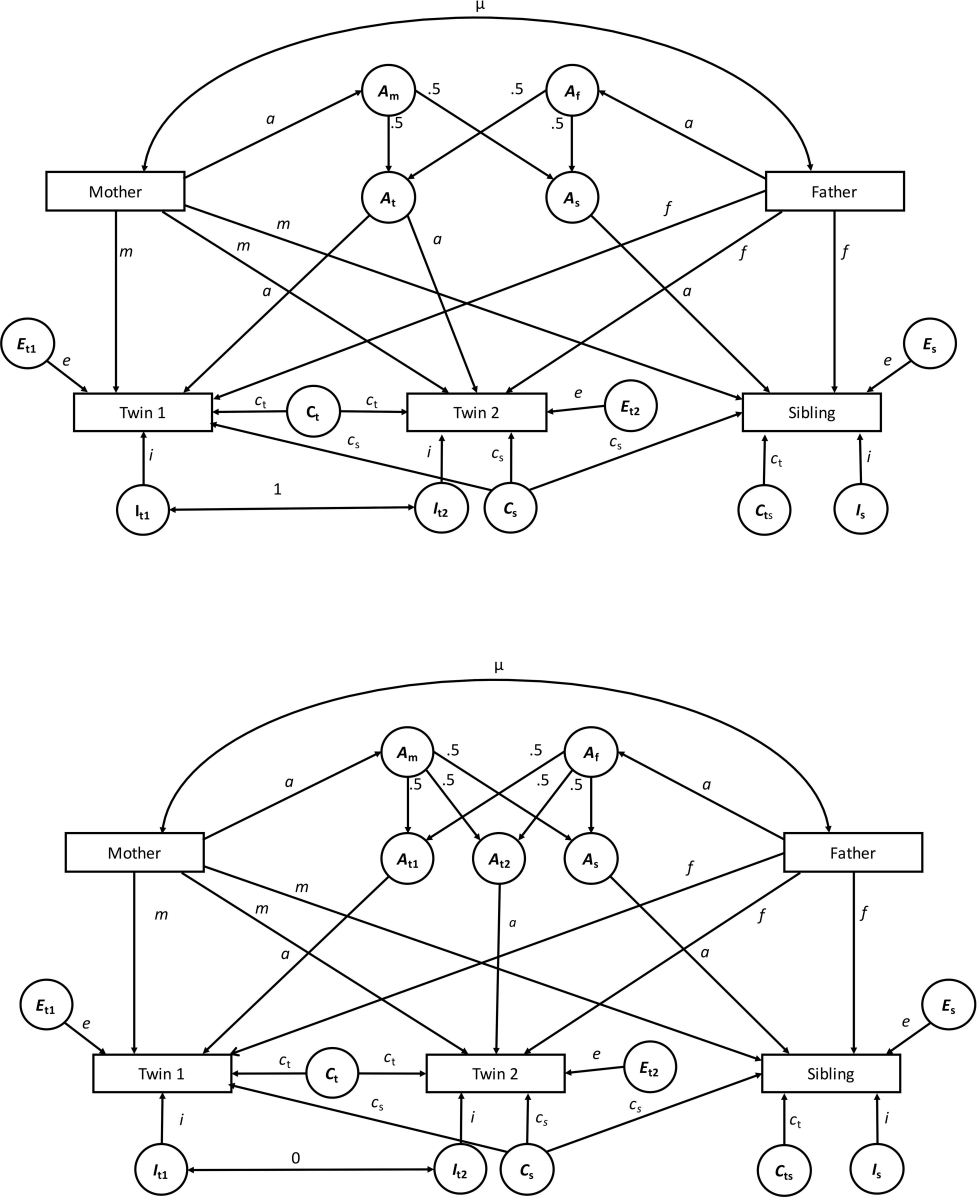
Extended Twin Family Model for monozygotic twins (upper part) and dizygotic twins (lower part). *a* = additive genetic effects; *i* = non-additive genetic effects due to epistasis; *e* = non-shared environmental effects including error of measurement; *m* = mother-specific environmental effects; *f* = father-specific environmental effects; *c*_s_ = sibling-specific shared environmental effects; *c*_t_ = twin-specific shared environmental effects; μ = assortative mating.

Since we had data not only for the twins, but also their parents and a sibling, we were able to derive our estimates from an Extended Twin Family Design (also Nuclear Twin Family Design; [36]). The ETFD has several advantages over the CTD (see [31], for a detailed overview) and allows a refined and more precise estimation of different genetic and environmental components. The full models for MZ and DZ twins are displayed in Fig 1. The ETFD separates additive genetic influences (*a*)―that is, effects due to different segregating genes given from parents to their offspring (50% from the father and 50% from the mother), which “add up” to affect phenotypic differences―from non-additive genetic influences, such as effects due to multiple interactions among different gene variants across different gene loci (epistasis, *i*). Whereas additive genetic effects are shared between relatives and act to increase their similarity as a function of their genetic relatedness, non-additive influences due to epistasis are completely shared only by MZ twins but not by other family relatives. They thus act to make MZ twins more similar but all other relatives less similar to each other. Both effects can be estimated in the presence of direct maternal (*m*) and paternal influences (*f*) from parents to offspring (i.e., the vertical transmission/socialization from parents to offspring that is shared by siblings and thus acts to make the children more similar) and non-shared environmental influences (i.e., environmental effects that are not shared by family members and thus act to make the children less similar, *e*, including error of measurement). This goes beyond the possibilities of the CTD and ameliorates the fact that, if certain effects are present but not taken into account in the model, estimates of variance components will be biased [31].

The inclusion of twins’ parents further enables the estimation of the contribution of assortative mating―the similarity of parents regarding the trait of interest (μ)―to the offspring’s similarity. Spousal similarity due to active assortment has been found to be especially pronounced for example in political attitudes (e.g., [37, 38]) and might also be true for POP and PI [39]. If assortative mating is present and not accounted for, shared environmental effects will be overestimated in a CTD, whereas additive genetic effects will be underestimated [31]. The model of twins and their parents thus enables to estimate the total variance component due to environmental transmission from both parents to offspring (*m*^*2*^ + *f*^*2*^ + 2*mf*μ).

A further advantage of the inclusion of the twins’ parents is that it allows for the estimation of the contribution of passive gene-environment covariation [40] to individual differences. Passive gene-environment covariation is the covariance between parents’ genetic makeup and the parental environment shared by the offspring, which means that the environment parents create for their offspring is correlated with the genetic makeup the children also inherit from their parents. For example, politically interested parents might discuss politics at home and encourage their children’s political activities, creating an environment that further fosters high political interest in their children. Passive gene-environment covariation can be estimated as the covariance between additive genetic effects and parental environment taking assortative mating into account: *a*^*2*^*m*(1 + μ) + *a*^*2*^*f*(1 + μ).

Besides adding the parents to the design, the additional inclusion of a full non-twin sibling of twins also increases the power to detect common environmental effects [41–43] and in addition makes it possible to disentangle environmental effects shared by non-twin siblings within an offspring generation (*c*_s_) from twin-specific, and thus age-related shared environmental influences (*c*_t_). For more advantages of the ETFD see [42]; for a detailed description of the parameters see [31].

Correlation analyses were based on scores corrected for linear age effects and sex differences and computed with SPSS 23. For the ETFD analyses, the unstandardized residual scores corrected for age and sex differences were entered into the models [44]. Four-group (2 zygosity groups × 2 age groups) structural equation models were conducted with AMOS 24 [45]. We identified the best fitting model via χ^2^-difference tests and the most parsimonious model by dropping the non-significant effects that did not lead to a decrease in model fit. Parameters are derived from the respective best-fitting, most parsimonious model. Differences between the cohorts were tested by setting the respective parameters equal and reviewing subsequent changes in model fit. Estimates were derived on the basis of full information maximum likelihood (FIML) procedures to analyze all available data and handle missing values due to a missing family member within specific families [46].

## Results

### Bivariate and family correlations

Bivariate correlations between the three variables ranged from rather low to high, the mean correlations (averaged over all family members) for SOP and POP were *r* = -.18, for SOP and PI *r* = -.13, and for POP and PI *r* = .45 (all single correlations were significant at *p* < .001). The univariate correlations between family members for the variables of interest are presented in Table 2 (as additional information, Twin-Cotwin correlations for male and female participants are presented in S1 Table). Since the degree of genetic relatedness between family members is known, comparing phenotypic correlations for different family members already gives a first indication regarding the effect of genes and the environment on individual differences in SOP, POP, and PI. In general, almost all correlations were significant. MZ twin correlations were highest, compared to all other correlations between family members that share on average only 50% of segregating genes (i.e. DZ twins, siblings, parents). Since MZ twins are 100% genetically related, the highest similarity of MZ twins compared to other family dyads indicates the presence of genetic effects. However, MZ correlations were below 1, which indicates non-shared environmental contributions to the variance. Furthermore, in the younger cohort, the DZ correlations tended to be more than half of the MZ correlations, which indicates the influence of shared environments. Indeed, and more specifically, in this cohort, the DZ correlations were on average higher than parent–child and sibling–twin correlations, which speaks for a twin-specific environmental influence that act to increase the similarity of same-aged siblings. Correlations between parents were also significant, indicating a medium high degree of assortative mating, except for a smaller correlation for PI.

**Table 2:** Family correlations for all dyads.

	Social Participation			Political Participation			Political Interest		
Dyads	*n*	*r* [95% CI]	*p*	*n*	*r* [95% CI]	*p*	*n*	*r* [95% CI]	*p*
C17									
MZ twin a and b	437	.696 [.623 - .757]	< .001	456	.500 [.402 - .586]	< .001	489	.504 [.420 - .577]	< .001
DZ twin a and b	508	.477 [.385 - .564]	< .001	527	.414 [.317 - .504]	< .001	552	.284 [.197 - .368]	< .001
Twin a and sibling	355	.337 [.230 - .442]	< .001	293	.191 [.071 - .306]	.001	395	.292 [.197 - .381]	< .001
Twin b and sibling	351	.273 [.168 - .379]	< .001	300	.225 [.113 - .337]	< .001	396	.162 [.053 - .267]	.001
Mother—twin a	782	.314 [.242 - .386]	< .001	842	.255 [.182 - .323]	< .001	970	.162 [.095 - .228]	< .001
Mother—twin b	771	.276 [.203 - .346]	< .001	842	.268 [.192 - .342]	< .001	964	.146 [.078 - .212]	< .001
Mother—sibling	292	.227 [.124 - .334]	< .001	258	.169 [.045 - .294]	.007	367	.141 [.042 - .237]	.007
Father—twin a	547	.353 [.266 - .440]	< .001	555	.215 [.130 - .301]	< .001	642	.125 [.047 - .201]	.002
Father—twin b	544	.264 [.176 - .353]	< .001	560	.253 [.172 - .333]	< .001	640	.119 [.042 - .197]	.003
Father—sibling	223	.169 [.028 - .305]	.001	193	.328 [.185 - .459]	< .001	278	.144 [.025 - .257]	.017
Parents	431	.426 [.340 - .509]	< .001	449	.452 [.369 - .532]	< .001	575	.128 [.048 - .211]	.002
C23									
MZ twin a and b	465	.568 [.473 - .654]	< .001	500	.509 [.432 - .583]	< .001	520	.526 [.451 - .597]	< .001
DZ twin a and b	410	.207 [.108 - .311]	< .001	426	.218 [.121 - .317]	< .001	448	.171 [.075 - .270]	< .001
Twin a and sibling	319	.155 [.040 - .266]	.006	201	.213 [.061 - .358]	.002	327	.272 [.170 - .367]	< .001
Twin b and sibling	316	.264 [.169 - .356]	< .001	205	.321 [.173 - .458]	< .001	329	.262 [.154 - .363]	< .001
Mother—twin a	750	.173 [.106 - .241]	< .001	798	.336 [.264 - .409]	< .001	894	.244 [.178 - .310]	< .001
Mother—twin b	734	.254 [.177 - .329]	< .001	785	.340 [.272 - .406]	< .001	893	.246 [.180 - .312]	< .001
Mother—sibling	264	.151 [.030 - .277]	.014	177	.303 [.145 - .448]	< .001	310	.321 [.194 - .420]	< .001
Father—twin a	402	.221 [.123 - .317]	< .001	431	.218 [.124 - .311]	< .001	490	.210 [.125 - .292]	< .001
Father—twin b	398	.323 [.223 - .418]	< .001	434	.192 [.096 - .291]	< .001	491	.177 [.084 - .268]	< .001
Father—sibling	155	.025 [-.130 - .187]	.755	109	.277 [.083 - .455]	.004	185	.262 [.125–394]	< .001
Parents	323	.425 [.318 - .523]	< .001	339	.376 [.274 - .474]	< .001	432	.263 [.172 - .348]	< .001

Note. Correlations are based on all biological family members. MZ = monozygotic; DZ = dizygotic. Twin a and b according to birth order. C17 = younger cohort; C23 = older cohort.

### Extended Twin Family Model estimates

In order to obtain stable parameter estimates, we identified the structural equation model (SEM) that fit the correlations between family members for each construct best while at the same time being the most parsimonious with regard to the number of parameters that had to be estimated. Therefore, we started with two baseline models in which either epistasis or sibling-specific shared environmental effects was set to zero (*i* = 0 or *c*_s_ = 0), because *i* and *c*_s_ cannot be estimated simultaneously in the present model. These non-nested models were descriptively compared by using the Comparative Fit Index (CFI) and Akaike’s Information Criterion (AIC). Larger CFIs and smaller AICs indicated a better model fit [47]. We then stepwise dropped non-significant parameters and checked for significant decrease in model fit as indicated by χ^*2*^-difference tests. A detailed account of model comparisons is presented in Tables A-C in S1 File.

In the best fitting, most parsimonious model for SOP, sibling-specific shared environmental effects were set to zero (baseline model, *χ*^*2*^ (45) = 113.133, *p* < .001; CFI = .935; RMSEA = .027). For POP, the model in which epistasis was set to zero in addition to sibling-specific effects fit the data best (*χ*^*2*^ (47) = 43.82, *p* = .61, *CFI* >.999, *RMSEA* < .001; Δχ^*2*^ = 2.603, Δdf = 2, *p* = .272). For PI, epistasis, sibling-specific, paternal and twin-specific shared environmental effects were dropped from the full model (*χ*^*2*^ (51) = 72.282, *p* = .03, *CFI* = 0.966, *RMSEA* = 0.014; Δχ^*2*^ = 7.961, Δdf = 6, *p* = .241). Standardized path estimates for model parameters are presented in Table 3, the resulting variance components in Table 4. Unstandardized path estimates and their respective confidence intervals are presented in S2 Table.

**Table 3:** Standardized path estimates for model parameters derived from the best fitting, most parsimonious ETFD model.

			Standardized model parameters						
		*a*	*i*	μ	*m*	*f*	*c*_t_	*c*_s_	*e*
SOP									
	C17	0.59	0.17	0.41	**0.18**	0.16	**0.45**	0.00	0.57
		(< .001)	(.273)	(< .001)	(< .001)	(< .001)	(< .001)	-	(< .001)
	C23	0.54	0.42	0.45	**0.11**	0.14	**0.16**	0.00	0.68
		(< .001)	(< .001)	(< .001)	(.001)	(.001)	(.360)	-	(< .001)
POP									
	C17	**0.50**	0.00	0.45	0.08	**0.08**	**0.45**	0.00	**0.70**
		(< .001)	-	(< .001)	(.058)	(.092)	(< .001)	-	(< .001)
	C23	**0.68**	0.00	0.39	0.12	**-0.12**	**0.00**	0.00	**0.72**
		(< .001)	-	(< .001)	(.009)	(.028)	(>.999)	-	(< .001)
PI									
	C17	0.70	0.00	**0.14**	0.03	0.00	0.00	0.00	0.71
		(< .001)	-	(< .001)	(.292)	-	-	-	(< .001)
	C23	0.67	0.00	**0.27**	0.12	0.00	0.00	0.00	0.71
		(< .001)	-	(< .001)	(< .001)	-	-	-	(< .001)

Note. *p* values in parentheses. Significant (*p* < .05) differences between cohorts are indicated by bold print. SOP = Social Participation; POP = Political Participation; PI = Political Interest; C17 = younger cohort; C23 = older cohort; *a* = additive genetic effects; *i* = non-additive genetic effects (epistasis); *e* = non-shared environmental effects including error of measurement; *m* = mother-specific environmental effects; *f* = father-specific environmental effects; *c*_s_ = sibling-specific shared environmental effects; *c*_t_ = twin-specific shared environmental effects; μ = assortative mating.

**Table 4:** Variance components derived from the best fitting, most parsimonious ETFD model.

		Standardized variance components						
		*a*^*2*^	*i*^*2*^	*a*^*2*^*m*(1 + μ) + *a*^*2*^*f*(1 + μ)	*m*^*2*^+*f*^*2*^+2*mf*μ	*c*_t_^*2*^	*c*_s_^*2*^	*e*^*2*^
SOP								
	C17	0.30	0.03	0.14	0.08	0.18	0.00	0.28
	C23	0.26	0.16	0.09	0.04	0.02	0.00	0.42
POP								
	C17	0.25	0.00	0.06	0.02	0.20	0.00	0.48
	C23	0.46	0.00	0.00	0.02	0.00	0.00	0.52
PI								
	C17	0.48	0.00	0.02	0.00	0.00	0.00	0.50
	C23	0.44	0.00	0.07	0.01	0.00	0.00	0.48

*Note*. SOP = Social Participation; POP = Political Participation; PI = Political Interest; C17 = younger cohort; C23 = older cohort; *a*^*2*^ = additive genetic component; *i*^*2*^ = non-additive genetic component; *e*^*2*^ = non-shared environmental component including error variance; *m*^2^ = variance due to maternal environmental transmission; *f*^2^ = variance due to paternal environmental transmission; *c*_s_^*2*^ = sibling-specific shared environmental component; *c*_t_^*2*^ = twin-specific shared environmental component; μ = assortative mating; *a*^*2*^*m*(1 + μ) + *a*^*2*^*f*(1 + μ) = passive gene-environment covariance; *m*^*2*^+*f*^*2*^+2*mf*μ = total parental environmental transmission

The best fitting models for SOP, POP, and PI yielded significant additive genetic effects (*a*). Besides this general pattern, cohort differences in the size of the genetic coefficients emerged (see Tables A-C in S2 File for detailed model comparisons). For SOP, non-additive genetic effects were larger for C23 than for C17 (explaining 16 vs. 3% of variance, respectively), even though this difference was not significant. A similar and significant age trend was visible for POP, where additive genetic effects were responsible for 25 vs. 46% of variance in C17 and C23 respectively, whereas for PI, additive genetic effects were similar for the two cohorts (44% and 48% of explained variance, respectively).

In addition to the additive genetic effects, environmental effects were significant in all models. They manifested most strongly in non-shared environmental effects (*e*), which make participants growing up in the same family less similar, but also include measurement error. These non-shared environmental effects explained between 28 and 52% of variance in SOP, POP, and PI, respectively. The effects tended to be larger in the older cohort for POP, whereas no significant cohort effects were detected for the other variables.

Influence of the shared environment, i.e. those environmental effects that make participants from the same family more similar to each other, was smaller than for the non-shared environment. We found evidence for effects of the environment that specifically act to increase the similarity of twins (*c*_t_) in C17 but not in C23 for SOP and POP, indicating that this environment might decrease in importance as the twins get older. Environmental transmission effects of the parents, i.e. parental socialization effects (*m*, *f*) were mostly small or non-significant in both age groups and for all variables, except for SOP in both cohorts and, interestingly, for POP and PI in C23.

Assortative mating (μ) was also present for all cohorts and variables providing evidence for the fact that with regard to their SOP, POP, and PI, partners were more similar than expected by chance. Coefficients for passive gene-environment covariation were positive and explained 14% of variance in C17 and 9% in C23 for SOP, 7% of variance in C23 for PI and 6% of variance for C17 in POP. This indicates that children in these age groups with a genetic predisposition to develop higher SOP, POP or PI, respectively, also grow up in environments that positively contribute to these traits.

## Discussion

The goal of our study was to disentangle genetic and environmental contributions to variance in SOP, POP, and PI with the help of a twin family design that yields information beyond the possibilities of the CTD and also allows for more confident estimates. Furthermore, our results go beyond previous studies by including a large twin sample from two age groups in crucial developmental periods that is representative of families with multiple children in Germany. Our results support previous studies showing that individual differences in POP and PI seem to be partly due to genetic differences [19], with up to almost 50% of phenotypic variation explained by genetic variation. Genetic effects were also found for SOP, albeit to a slightly smaller degree. This finding is especially notable since behavior genetic studies on social integration are rare. Genetic influences on the variance of these variables might be mediated by individual differences in a genetically affected inherent motive to affiliate with others in an organized way and to exert influence in a community. However, since it is likely that genetic differences exert their influences on individual differences in attitudes and behaviors in a rather complex way, other possible variables mediating the genetic effects, such as personality traits [48, 49] are promising candidates and should be investigated in future research.

As would be expected from the postulates of lifespan theories of gene expression and previous empirical findings [26], genetic effects were larger in the older cohorts for SOP (*a*^2^ plus *i*^2^) and POP (*a*^2^). For PI, however, estimates of genetic and environmental effects remained virtually the same across the two cohorts. This might be due to the fact that already in older adolescence, people have the freedom to show or not show interest in political affairs. It would thus be necessary to include even younger participants in order to investigate the onset of the genetic unfolding within the opportunities provided by individual environments (i.e., active gene-environment correlation). Furthermore, the results might also indicate that PI is a more trait-like prerequisite for continuing participation, developing earlier in life and thus paving the way for more intensive activities later on. However, since our analyses are solely based on cross-sectional data, we cannot make any claims regarding developmental processes. The speculation described above as well as all further discussions on developmental aspects need to be addressed by future research, preferably with longitudinal data and multiple measurement points that allow for the investigation of developmental and age-related processes.

As in previous studies on various other political traits [19], we found that individual experiences and exposure to environments not shared by family members are major contributors to individual differences in all three variables of interest, explaining a substantial part of variance in the respective variables (including variance due to error of measurement). Thus, individual factors such as peers, individual media exposure but also idiosyncratic experiences are candidates that might shape people’s participation and make them different from their family members.

Our study design allows for a refined and differentiated look at the contribution of environments shared by family members, so we could go beyond previous findings showing effects of the environment shared by twin siblings (but not by other family members) on individual differences in political attitudes in adolescence [23]. We found that the twin-specific shared environment but not the environment shared by the twins and the non-twin siblings plays a role for the variation of SOP and POP in younger participants. This might be shared friends, similar school and club contexts, and shared social networks. In addition, this includes environmental events of shorter duration that differentially impact children depending on their age. It might be worthwhile for future research to investigate what exactly these environments are made of and which developmental processes might play a role. Including habitational and emotional closeness of twins might also provide further insight. A limitation, however, is our relatively small sibling sample with a wide age range, thus the twin-specific effects warrant replication in future studies.

What is eye-catching in this matter is the fact that in almost all cases the impact of variation in the shared environment of any kind is smaller for variation in PI than for the two participation variables. This again might suggest that PI is developed earlier in life and the unfolding of variation in PI is less dependent on within-familial shared social influences in the investigated age. In line with other behavior genetic studies, parental environments had virtually no effects, speaking against the idea of parental socialization as one important source of individual differences in participation in politics and social life beyond genetic transmission. This finding, however, stands in contrast to adoption studies investigating the sources of individual differences in voting turnout and political candidacy, that find effects of pre-birth (i.e., genetic) and post-birth (i.e., environmental) differences to be both important and also similar in size [12, 50]. These studies, however, differ from ours in several aspects that might lead to the divergent findings. Different sample ages, contexts, variable operationalization (e.g., general mass participation vs. elite political behavior), analytical frameworks, and design assumptions complicate direct comparisons. This calls for a multi-method, multi-design approach to understand what exactly drives participation across the life span [50]. In addition, our study explicitly models passive gene-environment correlation (see below). In studies where this is not accounted for, these effects will instead lead to an overestimation of shared environmental effects [31].

For all variables, we found some effects for passive gene-environment correlation: The genetic makeup of the parents covaries with the environments they provide for their children which in turn might influence between-family differences in their children’s traits. Furthermore, since our model only tests shared parental effects affecting all siblings in the same way, the possibility that parental effects might affect siblings differently is not accounted for. Children might perceive or react to the same parental influences in a different way, this would manifest not as environmental effect shared by family members but rather as non-shared environmental effect [40]. Our results thus do not imply that parents do not play a role in their children’s social and political development, but rather suggest that direct parental influences, if at all, only marginally affect an increase in the similarity of offspring regarding POP, SOP, and PI.

Some limitations have to be noted when interpreting the presented findings which at the same time point to important directions for future research. Our study does not provide any information on the factors that mediate between genetic differences and the phenotypic expression in SOP, POP, and PI. Genetically influenced individual characteristics such as personality characteristics and cognitive abilities might be interesting candidates that have for example been shown to account for genetic variance in political attitudes [38, 51]. Including these variables goes beyond the scope of our study but should be addressed in future research to understand the genetic sources of variation in POP, SOP, and PI. Another issue is the operationalization we chose for our variables of interest. The POP index showed relatively low reliability, which might increase estimates of non-shared environmental influences and decrease estimates of other sources of variance due to lower correlations between family members. However, the unidimensionality and content validity (being designed to assess a rather hetereogeneous conglomerate of behaviors [1]) of the scale support its use in our study [14, 52]. Besides, our measure of SOP mixes different behaviors of social participation aimed at social integration in general irrespective of their underlying motivation aimed at social contact vs. social responsibility whereas previous studies have focused on civic duty or volunteerism in a narrower sense [14, 21]. Future studies should include other operationalizations of the construct that allow to disentangle indicators of SOP with regard to their motivation (social integration vs. responsibility for the community).

Despite these limitations, our study provides confident estimates for genetic and environmental effects on individual differences in three variables related to political and social integration. Our findings attest to the role of both genetic and environmental factors in shaping people’s investment in their political and social surroundings and also gives first ideas about the role of gene-environment interplay. As such, it provides the basis for a better understanding of SOP, POP, and PI at different ages and thus a starting point for developmental investigations.

## Supporting information

S1 Table Twin-cotwin correlations for all cohorts and constructs divided by participant sex. *Note*. MZ = monozygotic twins; DZ = dizygotic twins; C17 = younger cohort; C23 = older cohort; Values in bold print differ between the sexes at *p* ≤ .05.
(DOCX)

S2 Table Unstandardized path estimates and 95% confidence intervals for model parameters derived from the best fitting, most parsimonious ETFD model. *Note*. *p*-values in parentheses. 95% confidence intervals in square brackets. SOP = Social Participation; POP = Political Participation; PI = Political Interest; C17 = younger cohort; C23 = older cohort; *a* = additive genetic effects; *i* = non-additive genetic effects (epistasis); *e* = non-shared environmental effects including error of measurement; *m* = mother-specific environmental effects; *f* = father-specific environmental effects; *c*_s_ = sibling-specific shared environmental effects; *c*_t_ = twin-specific shared environmental effects; μ = assortative mating.
(DOCX)

S1 File **(Tables A-C). Model fitting results.** *Note*. Best fitting, most parsimonious model is indicated by bold print. *i* = non-additive-genetic effects; *f* = father-specific environmental effects; *m* = mother-specific environmental effects; *c*_s_ = sibling-specific shared environmental effects; *c*_t_ = twin-specific environmental effects.
(DOCX)

S2 File **(Tables A-C). χ**^*2*^**-difference tests for cohort model comparisons.** *Note*. For cohort comparisons, the baseline model *c*_s_ = 0 was used. *a* = additive genetic effects; *i* = non-additive epistasis; *e* = non-shared environmental effects including error of measurement; *m* = mother-specific environmental effects; *f* = father-specific environmental effects; *c*_s_ = sibling-specific shared environmental effects; *c*_t_ = twin-specific shared environmental effects; μ = assortative mating.
(DOCX)
