# Supplementary material for: On the genetic and environmental sources of social and political participation in adolescence and early adulthood
Source: PLoS One. 2018 Aug 24;13(8):e0202518. doi: 10.1371/journal.pone.0202518 (PMC6108469; doi:10.1371/journal.pone.0202518)
Supplement: S1 Table — Note. MZ = monozygotic twins; DZ = dizygotic twins; C17 = younger cohort; C23 = older cohort; Values in bold print differ between the sexes at p ≤ .05. (DOCX) [file pone.0202518.s001.docx]

**Supporting information S1**

**S1 Table. Twin-cotwin correlations for all cohorts and constructs divided by participant sex.**

|  | Social Participation | | |  | Political Participation | | |  | Political Interest | | |
| --- | --- | --- | --- | --- | --- | --- | --- | --- | --- | --- | --- |
| Dyads | *n* | *r*  [95% CI] | *p* |  | *n* | *r*  [95% CI] | *p* |  | *n* | *r*  [95% CI] | *p* |
| **C17** |  |  |  |  |  |  |  |  |  |  |  |
| MZ twin a and b |  |  |  |  |  |  |  |  |  |  |  |
| male | 189 | .710 [.585 - .805] | <.001 |  | 196 | **.361 [.213 - .509]** | **<.001** |  | 212 | .549 [.442 - .645] | <.001 |
| female | 248 | .683 [.598 - .760] | <.001 |  | 260 | **.589 [.477 - .686]** | **<.001** |  | 277 | .459 [.337 - .568] | <.001 |
| DZ twin a and b |  |  |  |  |  |  |  |  |  |  |  |
| male | **208** | **.326 [.180 - .484]** | **<.001** |  | 223 | .350 [.204 - .486] | <.001 |  | 231 | .279 [.136 - .412] | <.001 |
| female | **300** | **.571[.470 - .662]** | **<.001** |  | 304 | .466 [.349 - .570] | <.001 |  | 321 | .291 [.181 - .397] | <.001 |
| **C23** |  |  |  |  |  |  |  |  |  |  |  |
| MZ twin a and b |  |  |  |  |  |  |  |  |  |  |  |
| male | 184 | .598 [.475 - .706] | <.001 |  | 202 | .515 [.382 - .629] | <.001 |  | 211 | .563 [.449 - .666] | <.001 |
| female | 281 | .546 [.409 - .675] | <.001 |  | 298 | .510 [.414 - .599] | <.001 |  | 309 | .495 [.387 - .589] | <.001 |
| DZ twin a and b |  |  |  |  |  |  |  |  |  |  |  |
| male | 175 | .168 [.027 - .307] | .027 |  | 181 | .223 [.072 - .373] | .003 |  | 193 | .183 [.023 - .335] | .011 |
| female | 235 | .232 [.087 - .382] | <.001 |  | 245 | .216 [.090 - .342] | .001 |  | 255 | .138 [.013 - .269] | .028 |

*Note*. MZ = monozygotic twins; DZ = dizygotic twins; C17 = younger cohort; C23 = older cohort; Values in bold print differ between the sexes at *p* ≤ .0
