# Supplementary material for: On the genetic and environmental sources of social and political participation in adolescence and early adulthood
Source: PLoS One. 2018 Aug 24;13(8):e0202518. doi: 10.1371/journal.pone.0202518 (PMC6108469; doi:10.1371/journal.pone.0202518)
Supplement: S2 Table — Note. p-values in parentheses. 95% confidence intervals in square brackets. SOP = Social Participation; POP = Political Participation; PI = Political Interest; C17 = younger cohort; C23 = older cohort; a = additive genetic effects; i = non-additive genetic effects (epistasis); e = non-shared environmental effects including error of measurement; m = mother-specific environmental effects; f = father-specific environmental effects; cs = sibling-specific shared environmental effects; ct = twin-specific shared environmental effects; μ = assortative mating. (DOCX) [file pone.0202518.s002.docx]

**Supporting information S3**

**S3 Table. Unstandardized path estimates and 95% confidence intervals for model parameters derived from the best fitting, most parsimonious ETFD model.**

|  |  |  | Unstandardized model parameters | | | | | | |
| --- | --- | --- | --- | --- | --- | --- | --- | --- | --- |
|  |  | *a* | *i* | μ | *m* | *f* | *c*_t_ | *c*_s_ | *e* |
| SOP |  |  |  |  |  |  |  |  |  |
|  | C17 | 0.243 | 0.072 | 0.054 | 0.202 | 0.177 | 0.186 | 0.00 | 0.234 |
|  |  | (<.001) | (.273) | (<.001) | (<.001) | (<.001) | (<.001) | - | (<.001) |
|  |  | [0.186 - 0.300] | [-0.057 - 0.201] | [0.042 - 0.066] | [0.131 -0.273] | [0.041; 0.097 – 0.257] | [0.141 - 0.231] | - | [0.218 - 0.250] |
|  | C23 | 0.173 | 0.135 | 0.060 | 0.098 | 0.121 | 0.053 | 0.00 | 0.220 |
|  |  | (<.001) | (<.001) | (<.001) | (.001) | (.001) | (.360) | - | (<.001) |
|  |  | [0.122 - 0.224] | [0.082 - 0.188] | [0.048 - 0.072] | [0.039 - 0.157] | [0.048 - 0.194] | [-0.061 - 0.167] | - | [0.206 - 0.233] |
| POP |  |  |  |  |  |  |  |  |  |
|  | C17 | -0.409 | 0.00 | 0.456 | 0.067 | 0.066 | 0.394 | 0.00 | 0.092 |
|  |  | (<.001) | - | (<.001) | (.058) | (.092) | (<.001) | - | (<.001) |
|  |  | [-0.523 - -0.295] | - | [0.376 - 0.536] | [-0.002 - 0.136] | [-0.010 - 0.142] | [0.325 - 0.463] | - | [0.059 - 0.125] |
|  | C23 | -0.596 | 0.00 | 0.387 | 0.117 | -0.113 | 0.000 | 0.00 | 0.703 |
|  |  | (<.001) | - | (<.001) | (.009) | (.028) | (>.999) | - | (<.001) |
|  |  | [-0.708 - -0.484] | - | [0.295 - 0.479] | [0.029 - 0.205] | [-0.213 - -0.013] | [<-1 – > 1] | - | [0.664 – 0.742] |
| PI |  |  |  |  |  |  |  |  |  |
|  | C17 | 0.488 | 0.00 | 0.068 | 0.029 | 0.00 | 0.00 | 0.00 | 0.517 |
|  |  | (<.001) | - | (<.001) | (.292) | - | - | - | (<.001) |
|  |  | [0.453 - 0.523] | - | [0.029 - 0.107] | [-.024 - .082] | - | - | - | [0.490 - 0.544] |
|  | C23 | 0.487 | 0.00 | 0.135 | 0.125 | 0.00 | 0.00 | 0.00 | 0.529 |
|  |  | (<.001) | - | (<.001) | (<.001) | - | - | - | (<.001) |
|  |  | [0.450 - 0.524] | - | [0.092 - 0.178] | [0.068 - 0.182] | - | - | - | [0.502 - 0.556] |

*Note. p*-values in parentheses. 95% confidence intervals in square brackets. SOP = Social Participation; POP = Political Participation; PI = Political Interest; C17 = younger cohort; C23 = older cohort; *a* = additive genetic effects; *i* = non-additive genetic effects (epistasis); *e* = non-shared environmental effects including error of measurement; *m* = mother-specific environmental effects; *f* = father-specific environmental effects; *c*_s_ = sibling-specific shared environmental effects; *c*_t_ = twin-specific shared environmental effects; μ = assortative mating.
