# Supplementary material for: On the genetic and environmental sources of social and political participation in adolescence and early adulthood
Source: PLoS One. 2018 Aug 24;13(8):e0202518. doi: 10.1371/journal.pone.0202518 (PMC6108469; doi:10.1371/journal.pone.0202518)
Supplement: S1 File — (Tables A-C). Model fitting results. Note. Best fitting, most parsimonious model is indicated by bold print. i = non-additive-genetic effects; f = father-specific environmental effects; m = mother-specific environmental effects; cs = sibling-specific shared environmental effects; ct = twin-specific environmental effects. (DOCX) [file pone.0202518.s003.docx]

**Supporting information S1 file**

**S1 File. Table A. Model fitting results for Social Participation**

|  |  |  |  | Fit statistics |  |  |
| --- | --- | --- | --- | --- | --- | --- |
|  | χ²(df) | df | *p* | CFI | RMSEA | AIC |
| *i*=0 | 119.681 | 45 | <.001 | .928 | .029 | 189.681 |
| ***c*_s_=0** | **113.133** | **45** | **<.001** | **.935** | **.027** | **183.133** |
| *i=c*_s_*=*0 | 119.681 | 47 | <.001 | .930 | .028 | 185.681 |
| *i=c*_t_=0 | 142.351 | 47 | <.001 | .908 | .032 | 208.351 |
| *i=f=*0 | 149.438 | 47 | <.001 | .902 | .033 | 226.374 |
| *m=f=c*_s_*=c*_t_*=*0 | 257.307 | 51 | <.001 | .802 | .045 | 315.307 |
| *c*_s_*=c*_t_*=i=*0 | 142.351 | 49 | <.001 | .910 | .031 | 204.351 |
| *i=f=c*_t_*=c*_s_*=*0 | 168.374 | 51 | <.001 | .887 | .034 | 215.438 |

*Note*. Best fitting, most parsimonious model is indicated by bold print. *i* = non-additive-genetic effects; *f* = father-specific environmental effects; *m* = mother-specific environmental effects; *c*_s_ = sibling-specific shared environmental effects; *c*_t_ = twin-specific environmental effects

**S1 File. Table B. Model fitting results Political Participation**

|  | | Fit statistics | | | | |  |
| --- | --- | --- | --- | --- | --- | --- | --- |
|  | χ²(df) | | df | *p* | CFI | RMSEA | AIC |
| *i*=0 (ACE) | 43.41 | | 45 | .54 | 1 | .000 | 113.413 |
| *c*_s_=0 | 41.22 | | 45 | .63 | 1 | .000 | 111.220 |
| ***i*=*c*_s_=0** | **43.82** | | **47** | **.61** | **1** | **.000** | **109.823** |
| *c*_s_*=i*=*f*=0 | 52.98 | | 49 | .32 | .995 | .006 | 114.982 |
| *c*_s_*=i=m=*0 | 57.99 | | 49 | .18 | .989 | .009 | 119.986 |
| *c*_s_*=i=m=f=*0 | 65.86 | | 51 | .09 | .983 | .012 | 123.857 |
| *c*_s_=*c*_t_=*i*=0 | 58.18 | | 49 | .04 | .978 | .010 | 120.183 |
| *m*=*f*=*c*_s_=*c*_t_=0 | 74.55 | | 51 | .02 | .972 | .015 | 130.545 |
| *i*=*f*=0 | 52.93 | | 47 | .26 | .993 | .008 | 118.931 |
| *i*=*m*=0 | 57.99 | | 47 | .13 | .987 | .011 | 123.986 |
| *i*=*c*_t_=0 | 57.69 | | 47 | .14 | .987 | .011 | 123.694 |

*Note*. Best fitting, most parsimonious model is indicated by bold print. *i* = non-additive-genetic effects; *f* = father-specific environmental effects; *m* = mother-specific environmental effects; *c*_s_ = sibling-specific shared environmental effects; *c*_t_ = twin-specific environmental effects.

**S1 File. Table C. Model fitting results for Political Interest**

|  | Fit statistics | | | | |  |
| --- | --- | --- | --- | --- | --- | --- |
|  | χ²(df) | df | *p* | CFI | RMSEA | AIC |
| *i*=0 (ACE) | 70.24 | 45 | .009 | .960 | .017 | 140.241 |
| *c*_s_=0 | 64.320 | 45 | .031 | .969 | .015 | 134.320 |
| ***i*=*c*_s_=*f*=*c*_t_=0** | **72.282** | **51** | **.027** | **.966** | **.014** | **130.282** |
| *m*=*f*=*c*_s_=*c*_t_=0 | 89.07 | 51 | .001 | .939 | .019 | 147.067 |
| *c*_s_=*c*_t_=*i*=0 | 71.358 | 49 | .020 | .96 | .015 | 133.358 |
| *i*=*c*_s_=0 | 70.821 | 49 | .016 | .965 | .015 | 132.821 |
| *i*=*c*_s_=*f*=0 | 70.241 | 47 | .022 | .963 | .016 | 132.821 |
| *i*=*c*_s_=*f*=*c*_t_=*m*=0 | 89.067 | 53 | .001 | .943 | .018 | 143.067 |

*Note*. Best fitting, most parsimonious model is indicated by bold print. *i* = non-additive-genetic effects; *f* = father-specific environmental effects; *m* = mother-specific environmental effects; *c*_s_ = sibling-specific shared environmental effects; *c*_t_ = twin-specific environmental effects
