# Supplementary material for: On the genetic and environmental sources of social and political participation in adolescence and early adulthood
Source: PLoS One. 2018 Aug 24;13(8):e0202518. doi: 10.1371/journal.pone.0202518 (PMC6108469; doi:10.1371/journal.pone.0202518)
Supplement: S2 File — (Tables A-C). χ2-difference tests for cohort model comparisons. Note. For cohort comparisons, the baseline model cs = 0 was used. a = additive genetic effects; i = non-additive epistasis; e = non-shared environmental effects including error of measurement; m = mother-specific environmental effects; f = father-specific environmental effects; cs = sibling-specific shared environmental effects; ct = twin-specific shared environmental effects; μ = assortative mating. (DOCX) [file pone.0202518.s004.docx]

**Supporting information S2 file**

**S2 File. Table A. χ²-difference tests for cohort model comparisons; Social Participation.**

|  | Δχ² | Δdf | *p* |
| --- | --- | --- | --- |
| Equal *a* | 3.626 | 1 | .057 |
| Equal μ | 0.460 | 1 | .498 |
| Equal *m* | 4.800 | 1 | .028 |
| Equal *f* | 1.144 | 1 | .285 |
| Equal *c*_t_ | 9.831 | 1 | .002 |
| Equal *i* | 1.223 | 1 | .269 |
| Equal *e* | 1.847 | 1 | .174 |

*Note.* For cohort comparisons, the baseline model *c*_s_ =0 was used. *a* = additive genetic effects; *i* = non-additive epistasis; *e* = non-shared environmental effects including error of measurement; *m* = mother-specific environmental effects; *f* = father-specific environmental effects; *c*_s_ = sibling-specific shared environmental effects; *c*_t_ = twin-specific shared environmental effects; μ = assortative mating.

**S2 File. Table B. χ²-difference tests for cohort model comparisons; Political Participation.**

|  | Δχ² | Δdf | *p* |
| --- | --- | --- | --- |
| Equal *a* | 7.296 | 1 | .007 |
| Equal μ | 1.508 | 1 | .219 |
| Equal *m* | 0.357 | 1 | .550 |
| Equal *f* | 9.462 | 1 | .002 |
| Equal *c*_t_ | 7.468 | 1 | .006 |
| Equal *c*_s_ | 0.410 | 1 | .552 |
| Equal *e* | 11.075 | 1 | .001 |

*Note.* For cohort comparisons, the baseline model *i*=0 was used. *a* = additive genetic effects; *i* = non-additive epistasis; *e* = non-shared environmental effects including error of measurement; *m* = mother-specific environmental effects; *f* = father-specific environmental effects; *c*_s_ = sibling-specific shared environmental effects; *c*_t_ = twin-specific shared environmental effects; μ = assortative mating.

**S2 File. Table C. χ²-differences tests for cohort model comparisons; Political Interest.**

|  | Δχ² | Δdf | *p* |
| --- | --- | --- | --- |
| Equal *a* | 0.216 | 1 | .642 |
| Equal μ | 5.160 | 1 | .023 |
| Equal *m* | 2.436 | 1 | .119 |
| Equal *f* | 0.314 | 1 | .575 |
| Equal *c*_t_ | 1.117 | 1 | .291 |
| Equal *c*_s_ | 0.000 | 1 | >.999 |
| Equal *e* | 0.579 | 1 | .459 |

*Note.* For cohort comparisons, the baseline model *i*=0 was used. *a* = additive genetic effects; *i* = non-additive epistasis; *e* = non-shared environmental effects including error of measurement; *m* = mother-specific environmental effects; *f* = father-specific environmental effects; *c*_s_ = sibling-specific shared environmental effects; *c*_t_ = twin-specific shared environmental effects; μ = assortative mating
